# Comments on Bruun, D.M. *et al.* Community-Based Recreational Football: A Novel Approach to Promote Physical Activity and Quality of Life in Prostate Cancer Survivors. *Int. J. Environ. Res. Public Health* 2014, 11, 5557–5585—Time to Raise Our Game

**DOI:** 10.3390/ijerph110706842

**Published:** 2014-07-02

**Authors:** Daniel Parnell, Andy Pringle, Jim McKenna, Stephen Zwolinsky

**Affiliations:** Centre for Active Lifestyles, Leeds Metropolitan University, Leeds LS1 3HE, UK; E-Mails: a.pringle@leedsmet.ac.uk (A.P.); j.mckenna@leedsmet.ac.uk (J.McK.); s.zwolinksy@leedsmet.ac.uk (S.Z.)

Bruun and colleagues [1] provide a timely and thorough insight into the potential health opportunities on offer via the structural organisation of football associations, football clubs and the global grip of the beautiful game. Their extensive evaluation framework represents an important clarion call for those concerned with football-led health improvement. At the same time, it is wise to consider how this can be made realistic and relevant to those who may regard the football-led ‘concept’ as too alternative or even inappropriate, in the contemporary socio-political and economic context.

To meet current concerns, football-led health improvement interventions must be both effective and efficient, not least because budgetary restraints inevitably stimulate comparisons between different programmes and approaches. Importantly, advocates can now point to compelling research and evaluation evidence indicating that football-based interventions (a) reach and engage older men with complex health needs [2], (b) reduces participants’ alcohol consumption [3], (c) increases physical activity [3] and (d) produce significant reductions in weight [4]. Football clubs deliver these effects every day through established community outreach-programmes. 

Yet, as a relatively novel approach, football-based interventions need to continue showing their worth, making evaluation imperative for securing even on-going funding [1,4]. Evaluation is also essential for demonstrating cost-effectiveness and comparative cost-effectiveness. These themes need to become targets for subsequent evaluations because they will increasingly be the concerns of those responsible for Public Health spending. It is no longer wise, nor acceptable, to overlook the integration of evaluation into project planning and delivery. 

As Public Health agencies feel the drawn out sting of financial austerity, it is vital to draw on the work of Bruun and colleagues [1] and on the growing evidence base to rally policy makers, commissioners, researchers and applied practitioners, to generate better evidence and to respond to what exists so they raise their game. 
